# Efficacy of Handwashing with Soap and Nail Clipping on Intestinal Parasitic Infections in School-Aged Children: A Factorial Cluster Randomized Controlled Trial

**DOI:** 10.1371/journal.pmed.1001837

**Published:** 2015-06-09

**Authors:** Mahmud Abdulkader Mahmud, Mark Spigt, Afework Mulugeta Bezabih, Ignacio Lopez Pavon, Geert-Jan Dinant, Roman Blanco Velasco

**Affiliations:** 1 Department of Medical Microbiology and Immunology, College of Health Sciences, Mekelle University, Mekelle, Ethiopia; 2 Department of Family Medicine, CAPHRI School for Public Health and Primary Care, Maastricht University, Maastricht, Netherlands; 3 Department of Public Health, College of Health Sciences, Mekelle University, Mekelle, Ethiopia; 4 Catalan Institute of Health, Santa Coloma de Gramenet, Spain; 5 Department of Surgery, School of Medicine, Alcala University, Madrid, Spain; The Hospital for Sick Children, PAKISTAN

## Abstract

**Background:**

Intestinal parasitic infections are highly endemic among school-aged children in resource-limited settings. To lower their impact, preventive measures should be implemented that are sustainable with available resources. The aim of this study was to assess the impact of handwashing with soap and nail clipping on the prevention of intestinal parasite reinfections.

**Methods and Findings:**

In this trial, 367 parasite-negative school-aged children (aged 6–15 y) were randomly assigned to receive both, one or the other, or neither of the interventions in a 2 × 2 factorial design. Assignment sequence was concealed. After 6 mo of follow-up, stool samples were examined using direct, concentration, and Kato-Katz methods. Hemoglobin levels were determined using a HemoCue spectrometer. The primary study outcomes were prevalence of intestinal parasite reinfection and infection intensity. The secondary outcome was anemia prevalence. Analysis was by intention to treat. Main effects were adjusted for sex, age, drinking water source, latrine use, pre-treatment parasites, handwashing with soap and nail clipping at baseline, and the other factor in the additive model. Fourteen percent (95% CI: 9% to 19%) of the children in the handwashing with soap intervention group were reinfected versus 29% (95% CI: 22% to 36%) in the groups with no handwashing with soap (adjusted odds ratio [AOR] 0.32, 95% CI: 0.17 to 0.62). Similarly, 17% (95% CI: 12% to 22%) of the children in the nail clipping intervention group were reinfected versus 26% (95% CI: 20% to 32%) in the groups with no nail clipping (AOR 0.51, 95% CI: 0.27 to 0.95). Likewise, following the intervention, 13% (95% CI: 8% to 18%) of the children in the handwashing group were anemic versus 23% (95% CI: 17% to 29%) in the groups with no handwashing with soap (AOR 0.39, 95% CI: 0.20 to 0.78). The prevalence of anemia did not differ significantly between children in the nail clipping group and those in the groups with no nail clipping (AOR 0.53, 95% CI: 0.27 to 1.04). The intensive follow-up and monitoring during this study made it such that the assessment of the observed intervention benefits was under rather ideal circumstances, and hence the study could possibly overestimate the effects when compared to usual conditions.

**Conclusions:**

Handwashing with soap at key times and weekly nail clipping significantly decreased intestinal parasite reinfection rates. Furthermore, the handwashing intervention significantly reduced anemia prevalence in children. The next essential step should be implementing pragmatic studies and developing more effective approaches to promote and implement handwashing with soap and nail clipping at larger scales.

## Abstract 2

In a factorial cluster randomized controlled trial, Mahmud Abdulkader Mahmud and colleagues examine the efficacy of handwashing with soap and nail clipping on intestinal parasitic infections in school-aged children.

## Editors' Summary

**Background:**

Intestinal parasitic infections are common human infections, particularly in resource-limited countries, where personal hygiene and access to clean water and sanitation (disposal of human feces and urine) is often poor. Worldwide, more than a billion people are infected with soil-transmitted helminths—roundworms, tapeworms, and other parasitic worms that live in the human intestine (gut). And millions of people are infected with protozoan (single-celled) intestinal parasites that cause diseases such as amebiasis and giardiasis. Both helminths and protozoan parasites are mainly spread by the fecal-oral route. Infected individuals excrete helminth eggs and protozoan parasites in their feces, and in regions where people regularly defecate in the open, the soil and water supplies become contaminated with parasites. People then ingest the parasites by eating raw, unwashed vegetables, by not washing their hands after handling contaminated soil, or by drinking contaminated water. Mild infections with helminths rarely have symptoms, but severe infections can cause abdominal pain, diarrhea, and malnutrition. Protozoan parasites also cause diarrhea. Importantly, among children, who are particularly susceptible to parasitic infections, intestinal parasite infections may slow growth, affect school performance, and cause anemia.

**Why Was This Study Done?:**

Intestinal worm and protozoan infections can be treated with anthelmintic drugs and antibiotics, respectively. However, reinfection is often rapid, and, particularly in resource-limited countries, additional preventative measures are needed that do not rely on drugs (parasites can become drug-resistant) and that are sustainable with available resources. Given that intestinal parasitic infections usually spread through the fecal-oral route, the promotion of handwashing with soap and regular fingernail clipping might be one way to reduce intestinal parasite infection rates in low-income settings. Handwashing prevents other types of infection, and both unwashed hands and dirty, untrimmed nails are associated with high rates of parasite infection. Here, the researchers investigate whether handwashing with soap and nail clipping reduce intestinal reinfection rates by undertaking a factorial cluster randomized controlled trial (a study that compares outcomes in groups of people chosen at random to receive different combinations of two or more interventions) among school-aged children in northern Ethiopia.

**What Did the Researchers Do and Find?:**

The researchers assigned 367 parasite-negative school-aged children to receive a handwashing intervention, a nail clipping intervention, both interventions, or neither intervention for six months. For the handwashing intervention, fieldworkers visited each intervention household weekly, provided soap, encouraged all the household members to wash their hands with water and soap at key times, such as before meals and after defecation, and checked on the household’s use of soap. For the nail clipping intervention, the fieldworkers clipped the nails of children in the intervention households every week. After six months, parasite reinfection (primary outcome) and anemia (secondary outcome) in the participants were assessed by examining stool samples for parasites and by measuring hemoglobin levels, respectively. After adjustment for factors likely to affect reinfection such as latrine use and drinking water source, 14% of the children in the handwashing with soap groups (handwashing alone and handwashing plus nail clipping) were reinfected with parasites compared to 29% of the children in the no handwashing groups (nail clipping only or neither intervention). Similarly, 17% of the children in the nail clipping groups were reinfected compared to 26% in the no nail clipping groups. Finally, handwashing (but not nail clipping) significantly reduced the rate of anemia among the children.

**What Do These Findings Mean?:**

These findings show that handwashing with soap at key times decreased intestinal parasite reinfection rates by 68% and that weekly nail clipping reduced reinfection rates by 49% among school-aged Ethiopian children. Thus, these findings support the promotion of proper handwashing and weekly nail clipping as a public health measure to reduce parasite reinfection rates in resource-limited regions. However, although both interventions were “efficacious” under trial conditions that included intensive monitoring and follow-up, handwashing and nail clipping may not be “effective” interventions. That is, they may not work as well under real-life conditions. Moreover, because long-established personal hygiene and sanitation practices may be hard to change, large-scale implementation of these interventions might be expensive. The researchers call, therefore, for pragmatic studies to be undertaken to investigate the performance of these interventions under real-life conditions and for the development of effective approaches for widespread promotion of handwashing with soap and nail clipping.

**Additional Information:**

This list of resources contains links that can be accessed when viewing the PDF on a device or via the online version of the article at http://dx.doi.org/10.1371/journal.pmed.1001837.

- The US Centers for Disease Control and Prevention provides basic information about protozoan parasites and helminths; its Alphabetical Index of Parasitic Diseases provides more information about roundworms, tapeworms, giardiasis, amebiasis, and other intestinal parasites/parasitic infections; it also provides information about handwashing and about handwashing as a family activity
- The World Health Organization provides detailed information about intestinal worms, including a description of its current control strategy
- PARA-SITE is a multimedia resource provided by the Australian Society of Parasitology that provides detailed information about the biology of intestinal and other parasites
- KidsHealth, a site provided by the US-based non-profit Nemours Foundation, provides information for parents, kids, and teenagers about several intestinal parasites and about the importance of handwashing for parents, kids, and teenagers (in English and Spanish)

## Introduction

Intestinal parasitic infections are highly prevalent in the resource-limited regions of the world [1]. School-aged children are particularly susceptible to parasitic infections [1,2]. Both protozoan and helminthic infections correlate with unrecognized morbidities including growth deficits, malnutrition, and poor school performance [3]. Furthermore, intestinal parasitic infections are reported to be substantially linked with anemia in children. Intestinal parasitic infections can decrease food and nutrient intake, cause intestinal blood losses, and induce red blood cell destruction by the spleen [4,5].

The current strategy to control intestinal worm infections is periodic treatment of people at risk [6]. However, providing anthelminthic drugs systematically is difficult [7] and may increase potential drug resistance [3]. Furthermore, drug therapy alone only temporarily solves the problem, considering that reinfection occurs frequently in areas where intestinal parasitic infections are highly endemic [2].

To lower the dependency on a “drug only” approach and to enhance sustainability, complementary measures should be implemented [8–10] that are sustainable with available resources. Human hands are important vectors that carry disease-causing pathogens [11]. Therefore, handwashing is one of the most important interventions proven to effectively reduce the incidence of infectious diseases [12]. Handwashing, especially with soap, has been shown, for example, to be an effective preventive measure for diarrheal [13,14], and respiratory [14,15] diseases.

However, very little information on the impact of handwashing on intestinal parasitic infections and anemia is available, and existing evidence about whether handwashing is effective is inconclusive [16]. In addition to unclean hands, dirty and untrimmed nails have been associated with high parasite infection prevalence in observational studies [17,18]. However, there is no evidence for a potential beneficial effect of nail clipping on parasitic infections.

Since the fecal-oral route is the main dissemination pathway for parasitic infections, it is reasonable to suggest that promotion of handwashing with soap and fingernail clipping may reduce both the prevalence and intensity of intestinal parasite reinfections. These interventions can be done in low-income settings. We undertook a factorial randomized controlled trial to assess the effect of handwashing with soap and nail clipping on the prevalence of intestinal parasite reinfection, infection intensity, and prevalence of anemia among randomly assigned school-aged children within households in rural areas of northern Ethiopia.

## Methods

### Ethical Considerations

Ethical clearance was obtained from the institutional ethical review board of the College of Health Sciences, Mekelle University, Ethiopia. All participants and/or their guardians gave written informed consent/assent for participation. Information sheets were read to participants in the local language (Tigrigna), with explanations about the proposed home-based activities. Children who were diagnosed positive for intestinal parasitic infections at follow-up were treated with standard medication [19].

### Study Design

A 2 × 2 factorial clustered randomized trial was carried out in a rural area of northern Ethiopia to evaluate the impact of handwashing with soap and nail clipping on intestinal parasite reinfection rate (primary outcome), infection intensity (primary outcome; as measured by the arithmetic mean number of eggs per gram of stool), and anemia prevalence (secondary outcome) among randomly assigned school-aged children within households, after 6 mo of follow-up.

### Setting and Study Population

A scattered rural community within the northern Ethiopian Demographic and Health Surveillance site was selected based on the high prevalence of intestinal parasitic infections among school children [18]. A total of 216 households with at least one school-aged child (aged 6–15 y) were randomly selected using the Demographic and Health Surveillance household census as a sampling frame. In households with more than one school-aged child, two children were recruited randomly and were given the same intervention, resulting in a total number of 367 children.

Eligible children were aged 6–15 y, screened negative for intestinal parasitic infections either at baseline or after pre-trial anti-parasitic treatment, were planning to continue to reside in the same house for the study period, and had an informed consent signed by a parent or guardian. The exclusion criteria were being positive for intestinal parasites after pre-trial treatment or having a severe physical or mental disability.

### Randomization

**Fig 1:**
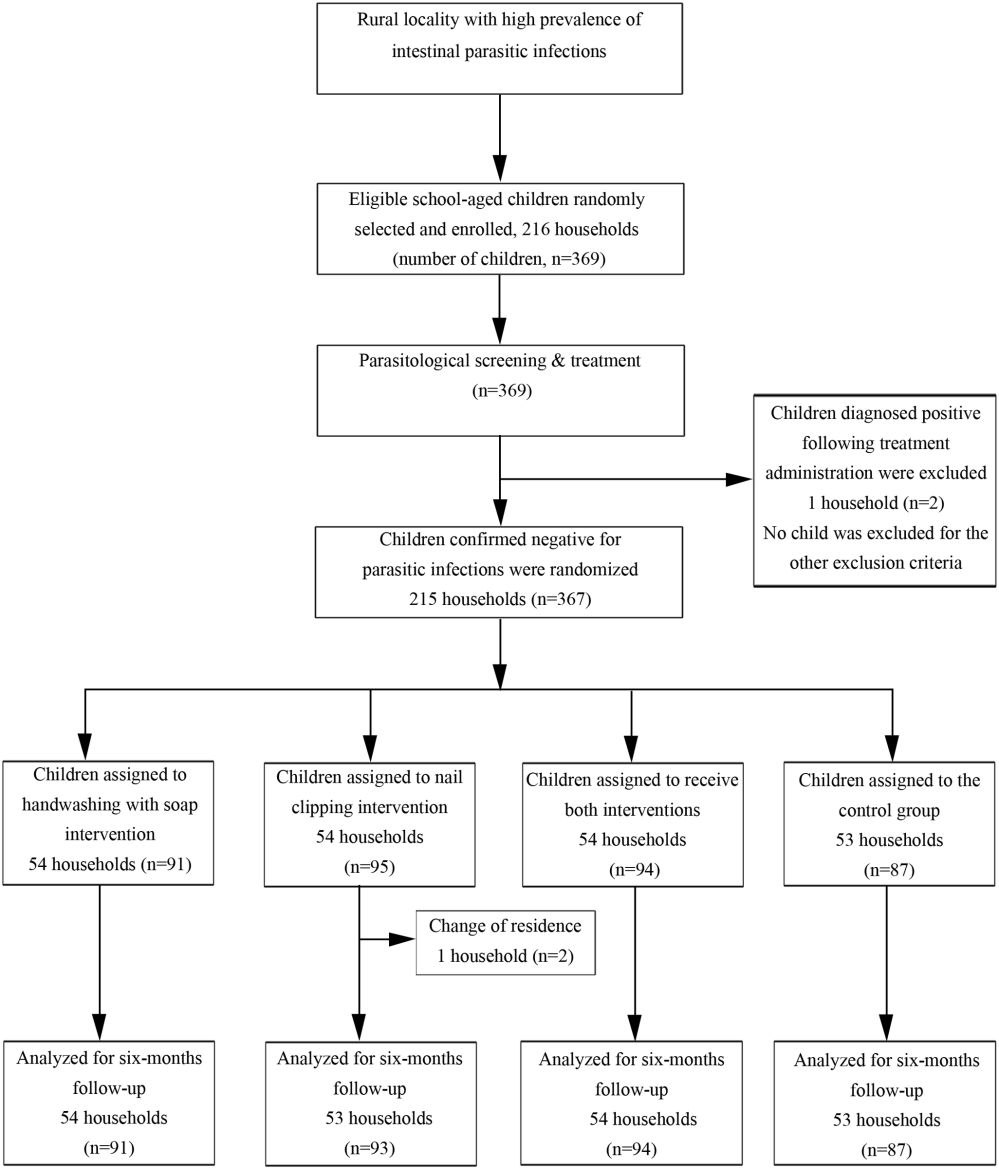
Trial profile: 2 × 2 factorial design.

Eligible children within the households were randomly assigned to receive both, one or the other, or neither of the interventions (Fig 1). One of the investigators who did not participate in recruiting the study participants randomly allocated the intervention groups using computer-generated random numbers in pre-prepared sealed, numbered envelopes. To facilitate blinding, the study was explained as an assessment of intestinal parasitosis among school-aged children, while the principal purpose of the trial was concealed. The assignment sequence was concealed from the researchers recruiting the study participants until interventions were assigned. Laboratory personnel were blinded to group assignments and to the assessment outcomes. Participating children (and their families) were aware of the intervention they received, but were blinded for the study hypothesis and the intervention(s) given to the other groups.

### Procedure

Following acquisition of signed informed consent, a series of parasitological screening steps—and when necessary parasite treatment steps—was carried out. Parasite-negative children were randomly assigned to handwashing with soap, nail clipping, or both interventions, or to continue with existing habits and practices (Fig 1). Children in each group were followed up for 6 mo (from June 1 to November 30, 2012).

### Interventions

#### Handwashing with soap

A total of eight fieldworkers were recruited to implement the intervention. Fieldworkers encouraged all individuals in the intervention household (who were old enough to understand) to wash their hands with water and soap before meals, after defecation, after playing on the ground, before preparing food, after cleaning an infant who had defecated, before feeding infants, and whenever their hands got unclean. Initially, fieldworkers provided 2–4 bars (120 g each) of plain soap per household, depending on size. Soap was regularly replaced throughout the study period. The provided soap was used exclusively for handwashing, and not for other purposes, as per the directives of the study.

Fieldworkers encouraged participants to wet their hands, lather them with soap, rub hands together for 45 s, and rinse the lather off with running water. Hands were dried with clean cloths prepared by the households. Children were instructed to hum a song to keep the time needed to rub their hands. Fieldworkers visited intervention households every week, for an average of 10–15 min each, to correct handwashing techniques and promote regular handwashing with soap at key times. Compliance was ascertained weekly by observing the size of the soap and the cleanliness of both hands, and by asking participants to demonstrate the handwashing procedure.

#### Nail clipping

Fieldworkers clipped the fingernails of children assigned to the nail clipping intervention on a weekly basis. New nail clippers, provided by the study, were kept by the fieldworkers and tagged with code numbers for each child in the intervention group for hygienic reasons. Nail clippers were replaced when necessary.

#### Control condition

Fieldworkers provided the control households with a regular monthly supply of sugar in an effort to preserve willingness to participate, but they gave no products that would be expected to affect handwashing and nail clipping behavior. They neither encouraged nor discouraged handwashing or nail clipping in control households, and visited both control and intervention households with equal frequency. Control households were frequently checked during the weekly visits for the presence of soap for handwashing and nail clippers to check for unintentional exposure of those in the control group to aspects of the experimental conditions (handwashing and nail clipping).

### Outcome Measures

The trial was stopped after 6 mo of follow-up. The primary outcome measures of our study were reduction in the prevalence and intensity of intestinal parasite reinfection for any parasitic infections identified among the children in the intervention groups. The planned primary outcome measure parasite reinfection rate was wrongly indicated as a secondary outcome in the initial registration of the trial (ClinicalTrials.gov, NCT01619254). The trial registration was corrected according the study protocol (S1 Protocol) on January 31, 2015. The secondary outcome measure was reduction in anemia prevalence.

### Sociodemographic Data Collection

Two separate structured questionnaires (child and household) were administered by the investigators in a local language during recruitment, which took place in April and May 2012, to provide information on demographics and personal hygiene and sanitation behaviors. Information on personal hygiene and sanitation behaviors at baseline was self-reported. The reported age of children was cross‐checked using baptism certificates, school records, local calendars, and information from parents.

### Parasitological Examination

Following the intervention, a thumb-sized fresh stool specimen was collected from all study participants. Children were brought to the school where our lab was established, and were provided with a clean, labelled plastic screw-top container (for sample collection), a plastic sheet (to catch the stool in the toilet), and an applicator stick (to transfer the sample). Stool specimens were analyzed by well-trained, blinded laboratory personnel, using direct saline wet mount, the formalin–ethyl acetate concentration technique [20], and the Kato-Katz technique (thick smear, 41.7 mg) [21]. Duplicate slides were prepared for each stool specimen and for each of the techniques used. For the Kato-Katz preparations, an average of the two samples was taken whenever there was a difference between the counts. Specimens were immediately processed, and the Kato-Katz and wet mount preparations were analyzed within 30 min to detect hookworm eggs and protozoan trophozoites (*Entamoeba histolytica/E*. *dispar* and *Giardia lamblia*, respectively). The remaining stool specimens were kept in 10% formalin and were examined using the concentration method within 2 h after collection. Kato-Katz preparations were reexamined after 72 h for the detection of helminth ova. A child was classified as reinfected if an infection was detected by any of the methods used.

The number of eggs for each helminth parasite detected was counted and multiplied by 24 to obtain the number of eggs per gram of feces (Vestegraard Frandsen group, Denmark). Ten percent subsamples of stool smears were reexamined for quality control purposes.

### Hemoglobin Survey

At baseline and at the end of the 6-mo follow-up, hemoglobin concentration was determined in finger prick blood using a HemoCue analyzer on site (HemoCue Hb 201z) [22]. Two microcuvette preparations were analyzed from a single blood specimen. The final measure was the mean of these two measurements. Technicians collecting and analyzing blood samples were trained for 1 d on machine operation before the actual data collection. The machines were checked on a daily basis using reference microcuvettes, as indicated by the manufacturer. Hemoglobin readings were adjusted for altitude, and anemia was defined for respective age and sex groups based on the World Health Organization cutoff values [23].

### Data Analysis

The primary hypothesis of the study was that handwashing with soap and nail clipping would significantly reduce the prevalence of intestinal parasite reinfection. Participants were analyzed according to the group to which they were randomized (intention to treat). We calculated a required sample size of 216 households using the formula for comparison of proportion of successes [24]; the households were evenly allocated into the intervention and control arms. The required sample size was calculated based on the following assumption: a prevalence of 72% [18], a minimum detectable difference of 20%, a power of 80%, a significance level of 0.05, and a 20% dropout rate. Sample size calculation was performed with an assumption of no interaction between the two factors (handwashing with soap and nail clipping). Separate sample size calculations were carried out based on target effect sizes for each of the interventions, and the larger sample size was taken as the trial sample size to enable the trial to be powered to detect the main effects of each intervention.

Statistical analysis was done using Stata 13.1. Our analysis focused on the main effects of the interventions (i.e., handwashing with soap versus not and nail clipping versus not), as is customary for studies with factorial designs, but the effects were also analyzed for the four intervention groups separately. Following the main effect analysis, effect modification was investigated by adding an interaction term to the regression models. Based on the nature of the study design (S1 Checklist), multilevel logistic regression models were used, taking into account the clustering of children in households, to investigate the efficacy of the interventions in reducing intestinal parasite reinfection rates and anemia prevalence, as represented by odds ratios (ORs) and 95% confidence intervals (CIs). The main effect analysis included both factors (handwashing with soap and nail clipping) in the same additive model. The main effects were adjusted for child sex, age, drinking water source, latrine use, handwashing with soap and nail hygiene at baseline, pre-treatment parasites, and the other factor in the additive model. For the secondary outcome, the effect was adjusted for sex, age, water source, latrine use, anemia at baseline, handwashing at baseline, nail hygiene at baseline, and the other factor in the additive model. Our analyses were adjusted for variables that were considered important potential confounders that were partly predefined and partly considered at peer review. Intraclass correlation coefficients (ICCs) based on the multilevel logistic regression models were computed as described by Rodriguez and Elo [25]. McNemar’s test was used to investigate associations between pre- and post-intervention parasite prevalence. The threshold for statistical significance was set at *p* < 0.05.

## Results

From the 369 school-aged children selected for the study, two were excluded before randomization and another two children were lost to follow-up because of a change in residential area (Fig 1). About 41% (*n =* 152) of the study participants were boys, and mean age was 10 y (standard deviation 2.6 y). At baseline, children in the four intervention groups were similar in terms of age and sex distribution, their personal hygiene and sanitation practices, and intestinal parasitic infection prevalence (Table 1).

**Table 1:** Baseline demographic, hygiene, and intestinal parasitosis characteristics by intervention group (*n =* 367).

Baseline Characteristic	Overall (*n =* 367)	Intervention Group			
		Handwashing with Soap (*n =* 91)	Nail Clipping (*n =* 95)	Handwashing with Soap and Nail Clipping (*n =* 94)	Control (*n =* 87)
**Sex**					
Male	150 (41)	38 (41)	39 (41)	38 (40)	35 (40)
Female	217 (59)	53 (59)	56 (59)	56 (60)	52 (60)
**Age**					
6–9 y	161 (44)	40 (44)	42 (44)	41 (44)	38 (44)
10–15 y	206 (56)	51 (56)	53 (56)	53 (56)	49 (56)
**Handwashing with soap**					
Yes	46 (13)	14 (15)	13(14)	9 (10)	10 (11)
No	321 (87)	77 (85)	82 (86)	85 (90)	77 (89)
**Handwashing before meal** ^**‡**^					
Yes	350 (95)	86 (95)	92 (97)	89 (95)	83 (95)
No	17 (5)	5 (5)	3 (3)	5 (5)	4 (5)
**Handwashing after defecation** ^**†**^					
Yes	50 (14)	11 (12)	13 (14)	14 (15)	12 (14)
No	317 (86)	80 (88)	82 (86)	80 (85)	75 (86)
**Nail hygiene**					
Trimmed	90 (25)	25 (27)	22 (23)	25 (27)	18 (21)
Untrimmed	277 (75)	66 (73)	73 (77)	69 (73)	69 (79)
**Drinking water source**					
Pipe	83 (23)	16 (18)	15 (16)	22 (23)	32 (37)
Hand pump	239 (65)	64 (70)	64 (67)	60 (64)	48 (55)
Wells or streams	45 (12)	11 (12)	16 (17)	12 (13)	7 (8)
**Latrine use**					
Yes	140 (38)	39 (43)	35 (37)	31 (33)	35 (40)
No	227 (62)	52 (57)	60 (63)	63 (67)	52 (60)
**Intestinal parasitosis**					
*E*. *histolytica*/*dispar*	109 (30)	31 (34)	27 (28)	28 (30)	23 (26)
*G*. *lamblia*	29 (8)	8 (9)	4 (4)	9 (10)	7 (8)
Hookworm	26 (7)	7 (8)	7 (7)	6 (6)	6 (7)
*Ascaris lumbricoides*	18 (5)	5 (5)	4 (4)	3 (3)	6 (7)
*Enterobius vermicularis*	36 (10)	9 (10)	10 (11)	8 (9)	9 (10)
*Hymenolepis nana*	52 (14)	13 (14)	13 (14)	13 (14)	13 (15)
Total parasitosis	267 (73)	72 (79)	64 (67)	67 (71)	64 (74)

Data are given as *n* (percent).

^‡^Using water only.

^†^Using water only or water and soap.

Pre-treatment prevalence of intestinal parasitic infections was high (73%) among the children, and both protozoans (*E*. *histolytica*/*dispar* and *G*. *lamblia*) and worms—for which the most important mode of transmission is fecal-oral—were observed (Table 1).

Households had a mean of 5.9 members (standard deviation 2.0). During the study, households assigned to the handwashing intervention received an average of 1.5 bars (120 g each) of soap per week; thus, about 4.3 g of soap was used per person per day in the intervention group. Throughout the course of the follow-up, no soap for handwashing and no nail clippers were observed in the control households. Almost all households (99%) in the handwashing group complied with the protocol, with only one household with two participating children that did not use the soap. All children assigned to the nail clipping intervention were available for the weekly nail clipping.

### Primary Outcomes

Table 2 provides the descriptive and multilevel logistic regression analysis results for the primary outcome intestinal parasite reinfection rate. The interaction between the interventions was investigated as a secondary analysis and was found to be not significant (*p* = 0.069). After 6 mo of follow-up, 14% (95% CI: 9% to 19%) of the children who received the handwashing with soap intervention were reinfected versus 29% (95% CI: 22% to 36%) of the children not receiving the handwashing with soap intervention (adjusted OR [AOR] 0.32, 95% CI: 0.17 to 0.62). Similarly, 17% (95% CI: 12% to 22%) of the children in the nail clipping group were reinfected versus 26% (95% CI: 20% to 32%) of the children not receiving the nail clipping intervention (AOR 0.51, 95% CI: 0.27 to 0.95). When looking at the four groups individually, reinfection occurred in 14% (13/91) of the children who received handwashing with soap only (AOR 0.19, 95% CI: 0.08 to 0.47), 14% (13/94) of the children who received both interventions (AOR 0.19, 95% CI: 0.08 to 0.48), 21% (20/95) of the children who received nail clipping only (AOR 0.32, 95% CI 0.14 to 0.73), and 38% (33/87) of the children who received no intervention. Relatively few children were infected with worms at baseline and follow-up. Therefore, we could not analyze our second primary outcome, infection intensity (differences in egg counts between the groups).

**Table 2:** Intestinal parasite reinfection rates at 6-mo follow-up (*n =* 367).

Handwashing with Soap	Nail Clipping		
	Yes	No	Margin
**Yes**	14%	14%	14%
	13 out of 94 children	13 out of 91 children	26 out of 185 children
	OR 0.24 (CI 0.10 to 0.55)	OR 0.25 (CI 0.11 to 0.57)	OR 0.36 (CI 0.20 to 0.66)
	AOR 0.19 (CI 0.08 to 0.48)	AOR 0.19 (CI 0.08 to 0.47)	AOR 0.32 (CI 0.17 to 0.62)
**No**	21%	38%	29%
	20 out of 95 children	33 out of 87 children	53 out of 182 children
	OR 0.42 (CI 0.20 to 0.88)		
	AOR 0.32 (CI 0.14 to 0.73)	OR 1 (Ref)	OR 1 (Ref)
**Margin**	17%	26%	
	33 out of 189 children	46 out of 178 children	
	OR 0.59 (CI 0.33 to 1.03)	OR 1 (Ref)	
	AOR 0.51 (CI 0.27 to 0.95)		

Crude ORs and AORs are for comparisons of the intervention with the control. AORs are adjusted for sex, age, drinking water source, latrine use, pre-treatment parasites, handwashing with soap at baseline, nail clipping at baseline, and the other factor in the additive model. Interaction between the interventions in the adjusted model was not significant, *p* = 0.069. The ICC in the adjusted model was 0.14 without the interaction and 0.13 with the interaction.

### Secondary Outcome

Descriptive and multilevel logistic regression analysis results for the secondary outcome are provided in Table 3. The interaction between the interventions was found to be not significant (*p* = 0.814). At the end of the trial, 13% (95% CI: 8% to 18%) of the children receiving the handwashing intervention were anemic versus 23% (95% CI: 17% to 29%) of the children not receiving the handwashing intervention (AOR 0.39, 95% CI: 0.20 to 0.78). Similarly, 14% (95% CI: 10% to 18%) children receiving the nail clipping intervention were anemic versus 21% (95% CI: 17% to 25%) of the children not receiving the nail clipping intervention; however, the observed difference was not statistically significant (AOR 0.53, 95% CI: 0.27 to 1.04). When looking at the four groups individually, anemia was observed in 14% (13/91) of the children who received handwashing with soap only (AOR 0.37, 95% CI: 0.15 to 0.91), 12% (11/94) of the children who received both interventions (AOR 0.21, 95% CI: 0.08 to 0.58), 17% (16/95) of the children who received nail clipping only (AOR 0.49, 95% CI: 0.21 to 1.19), and 29% (25/87) of the children who received no intervention.

**Table 3:** Anemia prevalence at 6-mo follow-up (*n =* 367).

Handwashing with Soap	Nail Clipping		
	Yes	No	Margin
**Yes**	12%	14%	13%
	11 out of 94 children	13 out of 91 children	24 out of 185 children
	OR 0.22 (CI 0.08 to 0.58)	OR 0.38 (CI 0.16 to 0.92)	OR 0.40 (CI 0.21 to 0.78)
	AOR 0.21 (CI 0.08 to 0.58)	AOR 0.37 (CI 0.15 to 0.91)	AOR 0.39 (CI 0.20 to 0.78)
**No**	17%	29%	23%
	16 out of 95 children	25 out of 87 children	41 out of 182 children
	OR 0.51 (CI 0.22 to 1.20)		
	AOR 0.49 (CI 0.21 to 1.19)	OR 1 (Ref)	OR 1 (Ref)
**Margin**	14%	21%	
	27 out of 189 children	38 out of 178 children	
	OR 0.59 (CI 0.33 to 1.03)	OR 1 (Ref)	
	AOR 0.53 (CI 0.27 to 1.04)		

Crude ORs and AORs are for comparisons of the intervention with the control. AORs are adjusted for sex, age, drinking water source, latrine use, handwashing with soap at baseline, nail hygiene at baseline, anemia at baseline, and the other factor in the additive model. The interaction between the interventions in the adjusted model was not significant, *p* = 0.814. The ICC in the adjusted model was 0.26, regardless of whether the interaction was included in the model or not.

## Discussion

The purpose of this trial was to evaluate the impact of two simple public health interventions (handwashing with soap and fingernail clipping) on the risk of intestinal parasite reinfection and anemia among school-aged children.

Our interventions of handwashing with soap and weekly nail clipping for children with no intestinal parasites at baseline demonstrated a significant reduction in intestinal parasite reinfection rates at 6 mo. Children who received handwashing with soap at critical times were 68% less likely to be reinfected by intestinal parasites than children left to continue with existing habits and practices. Similarly, children whose nails were cut on a weekly basis were 49% less likely to be reinfected by intestinal parasites than children not receiving the nail clipping intervention. The unadjusted difference in intestinal parasite reinfection rate was not statistically significant for the nail clipping versus no nail clipping groups. Regarding anemia, children who received handwashing with soap were 61% less likely to be anemic than children who did not receive this intervention. However, anemia rates were not significantly reduced in the children who received the nail clipping intervention compared to those who did not.

Several observational studies have indicated the impact of handwashing on the prevention of intestinal parasitic infections [26–29]. A case-control study conducted in Viet Nam demonstrated a significantly reduced risk of *E*. *histolytica* infection among individuals who frequently washed their hands with soap [28]. A longitudinal cohort study by Monse and colleagues [29] demonstrated decreased rates of reinfection with soil-transmitted helminthes among school children who washed their hands with soap. Most of the studies did not take into account whether soap was effectively used. They used self-reported handwashing behavior as their exposure measure, a major methodological weakness that was addressed in the present study. Furthermore, to our knowledge, no randomized control trials have been conducted to address the causal impact of handwashing with soap and nail clipping on intestinal parasitic infections.

Significant reduction in anemia prevalence among children was reported from an interventional study that integrated handwashing and dietary modification interventions [30]. The confounding effects of deworming and dietary modification among the intervention group make identification of the specific component responsible for the reported improvements difficult. Furthermore, none of these the studies was designed to allow causal inference.

In addition to the immediate benefits for the improvement of the health of children under consideration, proper handwashing with soap and weekly trimming of fingernails can reduce the output of infective stages in feces that results in the contamination of the environment, and hence can reduce infection transmission in the community [3,31]. To be sure about this hypothesis, however, pragmatic trials involving a larger community than those who received the intervention in the present study are needed. Interventions involving handwashing are also documented to have a lasting pedagogical effect by decreasing infectious illness and hence decreasing school absenteeism [32].

### Strengths and Weaknesses

Our study demonstrated causal relationships between hand hygiene and infection and anemia among school-aged children. Although our data showed that handwashing and nail clipping were efficacious, our trial included intense follow-up and monitoring that involved a high human resource investment. Changing the long-established habitual and culturally embedded practices of personal hygiene and sanitation among the children and the households might require methods that would make large-scale implementations of such interventions more expensive. Furthermore, as in any other efficacy study, intervention benefits were assessed under specific conditions, which might limit the generalizability of the results both to clusters and individual participants and overestimate the intervention effects when implemented under usual circumstances.

Since labor is relatively cheap in Ethiopia and other low-income countries, national house-to-house education campaigns might be organized to promote handwashing with soap at key times and weekly nail clipping. Furthermore, handwashing and nail clipping interventions can also be integrated into the existing community health programs (the Health Extension Program, Demographic and Health Surveillance sites, and the Health Development Army network) in the country that reach inaccessible, impoverished populations through house-to-house visits as their outreach activities. Soap was freely provided in this study, and provision of free soap to all impoverished households might not be feasible for large-scale implementations. The next essential step, obviously, should be implementing pragmatic studies that investigate the performance of the interventions under circumstances that more closely approach real and usual conditions, and developing more effective approaches to promote and implement handwashing with soap and nail clipping at a larger scale.

### Conclusion

Our data showed that regular handwashing with soap and nail clipping are efficacious in preventing intestinal parasitic reinfections and thereby deliver health benefits to school-aged children at risk. Proper handwashing and weekly nail clipping may be considered for widespread implementation as a public health measure across societies of resource-limited regions to reduce infection transmission.

## Supporting Information

S1 Data Raw data file. (XLSX)

S1 Checklist CONSORT Extension for Cluster Trials Checklist. (DOCX)

S1 Protocol Factorial randomized controlled trial protocol. (PDF)

S1 Table Pre- and post-intervention prevalence of *E*. *histolytica* among school-aged children. (DOCX)

S2 Table Pre- and post-intervention prevalence of *G*. *lamblia* among school-aged children. (DOCX)

S3 Table Pre- and post-intervention prevalence of hookworm among school-aged children. (DOCX)

S4 Table Pre- and post-intervention prevalence of *En*. *vermicularis* among school-aged children. (DOCX)

S5 Table Pre- and post-intervention prevalence of *H*. *nana* among school-aged children. (DOCX)

S6 Table Pre- and post-intervention prevalence of *A*. *lumbricoides* among school-aged children. (DOCX)

## Abbreviations:

AOR: adjusted odds ratio
ICC: intraclass correlation coefficient; OR, odds ratio
OR: odds ratio
