# Supplementary material for: Efficacy of Handwashing with Soap and Nail Clipping on Intestinal Parasitic Infections in School-Aged Children: A Factorial Cluster Randomized Controlled Trial
Source: PLoS Med. 2015 Jun 9;12(6):e1001837. doi: 10.1371/journal.pmed.1001837 (PMC4461173; doi:10.1371/journal.pmed.1001837)
Supplement: S1 Protocol — (PDF) [file pmed.1001837.s003.pdf]

Hand washing with soap and nail clipping: effect on intestinal parasitic infection in school-aged children: a factorial randomised controlled trial

The study design shall be a factorial randomised controlled trial, whereby different intervention packages will be randomised among households with children recruited for the survey.

A total of 216 households with at least one school-aged child (aged 6-15) will be randomly selected by systematic random sampling method. Selection of households will be made based on the DHSS (Demographic Health Surveillance Site) baseline census. Hand-washing with soap intervention will be implemented at household level, using selected child (aged 6-15) within the household as study unit. If there is more than one child in the age group per randomised household two children will be selected by simple lottery method and will be included in the trial. Reported age of a child will be cross-checked using school- records, baptism certificates, local calendars and birth certificates (if available).

Each intervention and control group will have a fixed number of participants, and will be subjected to only one of the study interventions. Children in each group will be followed-up for 6 months and parasitological and haemoglobin data will be collected following intervention. Different intervention packages shall be evaluated separately (the primary comparisons are the main effects), in combination and also against the control groups (groups with standard habits and practices).

Sample size determination

Using the formula for 'comparison of proportion of successes' and assuming equal groups; based on data collected previously in the study area on prevalence of intestinal parasitosis among the age-group (72.2% -data from previous prevalence study in the area) and assuming prevalence reduction of 20%; taking a power of 80% and assuming a significance level of 0.05; the sample size per group (intervention and control) will be 90 households.

$$n = \frac{\left\{\begin{matrix}\text{success x failure}\\ \text{on treatment A}\end{matrix}\right\} + \left\{\begin{matrix}\text{success x failure}\\ \text{on treatment B}\end{matrix}\right\}}{(\text{success on A} - \text{success on B})^2} \text{ x 'magic number'}$$

Sample size = 88 (90); 90 households in intervention and 90 household in the control arm (n=180). Considering 20% loss-to-follow up = 180 + 36 = 216 households.

		Nail clipping (NC)		
		Yes	No	Total
Hand washing with soap (HW)	Yes	54	54	108
	No	54	54	108
		108	108	216

Requirement of parasite free children

Prior to random allocation to the intervention and control arms and following acquisition signed informed consent, children will be screened for intestinal parasitosis. Children screened positive for intestinal parasitic infections will be treated using standard treatment regimen. Parasite negative children in the respective households will be randomly assigned into different intervention and control groups and randomisation will be by sealed envelope method.

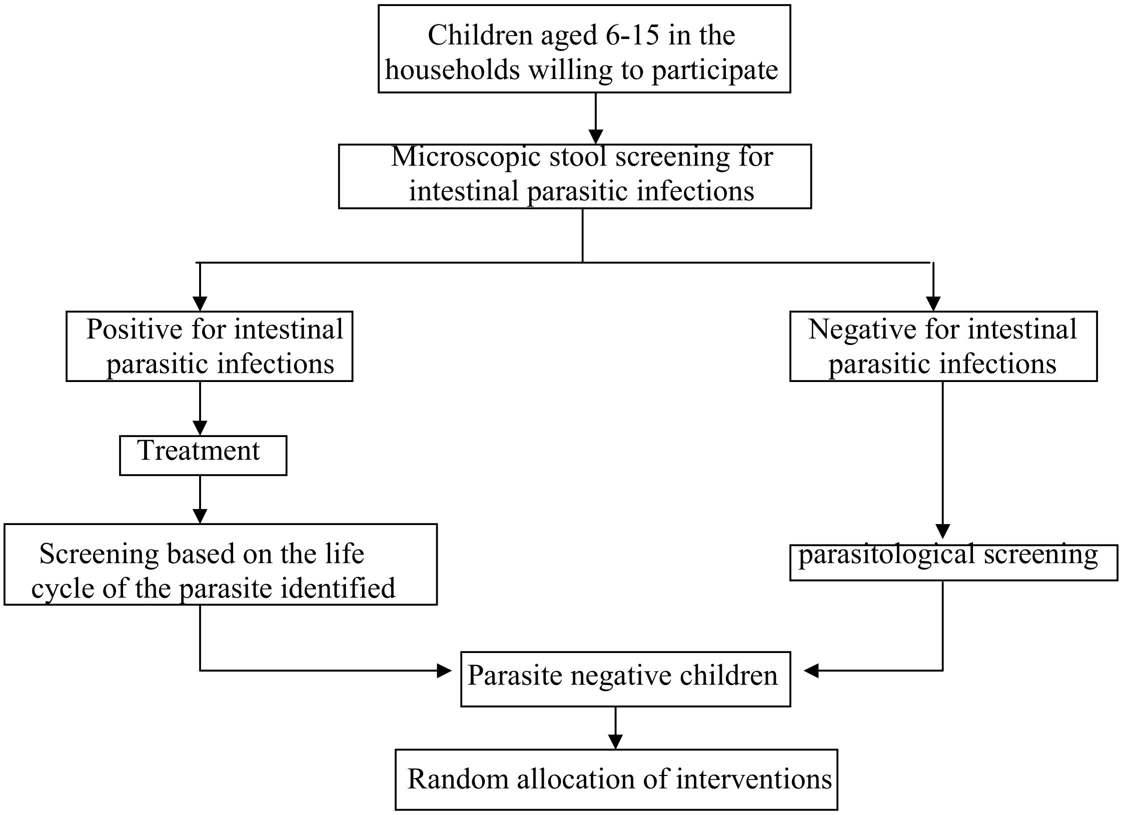

Parasitological examination

- A thumb sized fresh stool specimens will be collected on the spot from all the study subjects.
- Children will be provided with:
    - clean labelled plastic screw-top containers (for sample collection)
    - plastics sheet (to catch the stool in the toilet)
    - applicator stick (to transfer the sample).

Stool analysis will be done using:

- Direct saline wet mount
- Formalin ethyl-acetate concentration technique
- Kato-Katz technique (thick smear 41.7mg)

- Duplicate slides will be prepared for each stool specimen and for each of the techniques used.
- 10% of randomly selected stool smears will be re-examined for each sample collected for quality control purposes.

Sociodemographic data collection

Two separate structured questionnaires (child and household) will be prepared and administered in a local language during recruitment to generate information on the demographic and personal hygiene and sanitation behaviours.

Concealment and blinding

Children and household assignment sequence will be concealed. Researchers assessing the outcomes will be blinded to group assignments. Lab personnel will be blinded to group assignment and to the assessment outcomes. Participating children and their families can be aware of the intervention they receive but will be blinded for the study hypothesis and the intervention(s) given to the other groups. The study will be explained as an assessment of intestinal parasitosis, anaemia and malnutrition among school-aged children, while the principal purpose of assessing the impact of hand washing and nail clipping will be concealed.

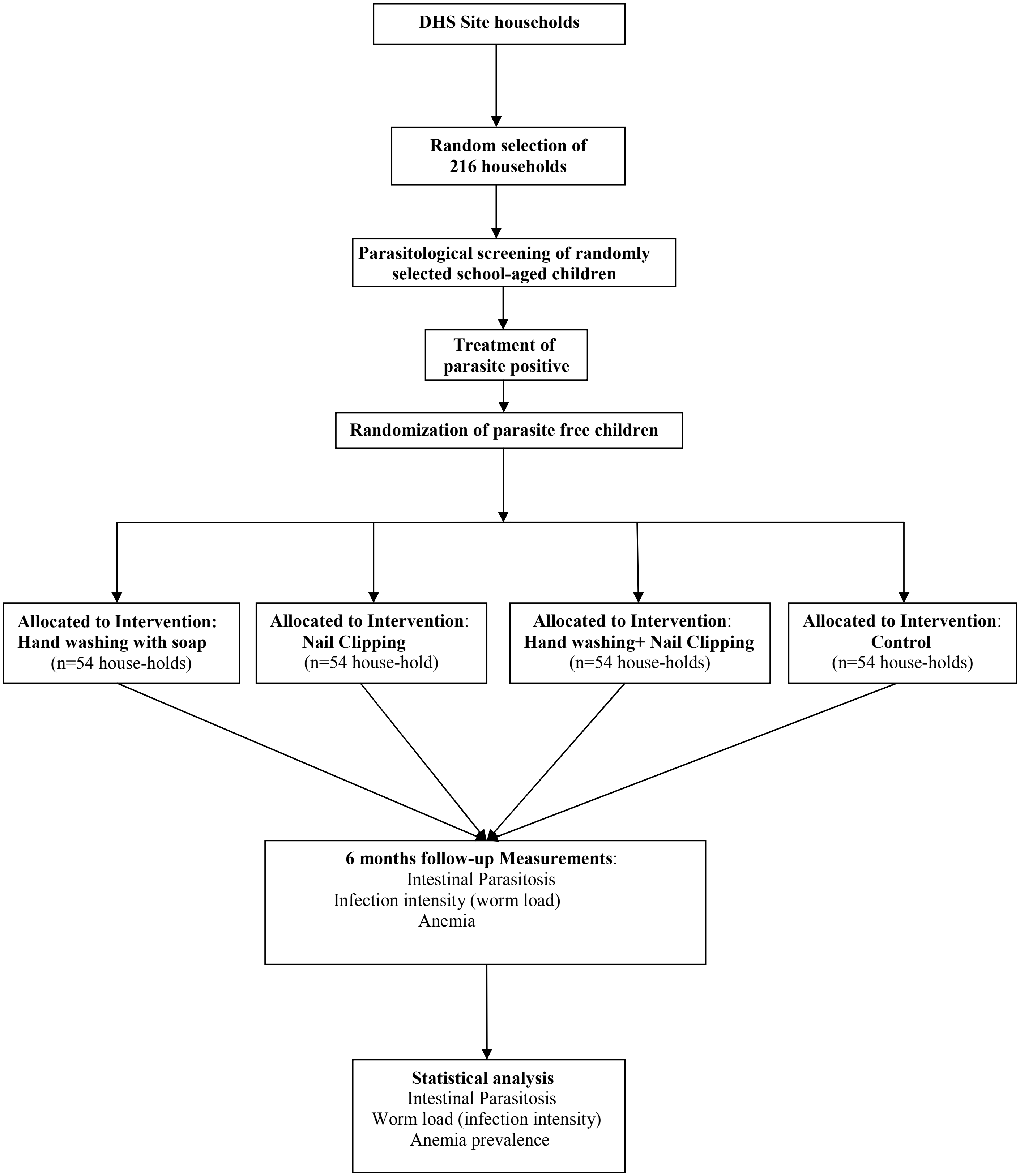
DHS Site households
Random selection of 216 households
Parasitological screening of randomly selected school-aged children
Treatment of parasite positive
Randomization of parasite free children
Allocated to Intervention: Hand washing with soap (n=54 house-holds)
Allocated to Intervention: Nail Clipping (n=54 house-hold)
Allocated to Intervention: Hand washing+ Nail Clipping (n=54 house-holds)
Allocated to Intervention: Control (n=54 house-holds)
6 months follow-up Measurements: Intestinal Parasitosis Infection intensity (worm load) Anemia
Statistical analysis Intestinal Parasitosis Worm load (infection intensity) Anemia prevalence

Specific Aim I: Evaluate the impact of hand washing with soap and hand nail clipping on parasite re-infection rates, intensity and self-reported illness.

Objective and Rationale: Effective preventive tools serve in decreasing the burden of entroparasite infections. Periodic drug administration, proper hygiene and sanitation are known to keep the levels of infection below those associated with morbidity, and hence improve the health and development of individuals, especially of children. The fact that parasitic infections impair children's growth and development is of a public health concern.

Although treatment reduces both transmission and infection rates of intestinal parasitic infections, whether this intervention measure is sustainable solution is not clear as rapid re-infection usually occurs. There is also emerging evidence that concurrent worm infections may have impacts on child development and nutritional status. The potential impacts of hand hygiene promotion activities on intestinal parasitosis and illness among children merit extensive investigation.

Methodology: Parasitological analysis will be carried out and children diagnosed positive for intestinal parasitic infections will be treated and recruited for the study. A detailed household listing and identification of households with children under investigation will be carried out. Upcoming allocation sequence will be concealed from participants and field workers, and households will be randomized to receive either: (a) hand washing with soap; or (b) hand nail clipping; or (c) both interventions; or (d) neither of the intervention packages. Children assigned to intervention arms of hand washing with soap and finger nail clipping will be provided with soap and nail clippers, respectively.

Fieldworkers will be trained on approaches to promote hand washing and hand nail clipping, standard procedure of hand washing with soap, data collection and reporting. Fieldworkers will visit households on a weekly basis throughout the study period to encourage hand washing, nail clipping and to record incidence of clinical symptoms. Same regular visits will be made to the control households. Sociodemographic data will be collected prior to the initiation of the study using structured questionnaire.

Data collection and processing: A series of parasitological screening activities will be done after completion each treatment phase based on the life cycle of each indentified parasite species until parasite positive children are screened negative. Children who received hand washing with soap will be compared with children who received no hand washing and children under nail clipping intervention will be compared against the non clipping group. Differences of impact in different subgroups and possible interactions between the different intervention activities (hand washing with soap and nail clipping) will be analysed.

The aim of the study and procedures employed will be discussed with the local administrative leaders and health authorities, and with parents and children participating in the study.

Quality of the tests and accuracy of egg counts of each parasitological analysis period will be assessed by screening a random sample of 10% of the slides for a second time by a well-experienced laboratory technologists.

Anticipated results: The primary outcome measures will be:

- the proportion of children with reduced intestinal parasitosis re-infection rates in the intervention and control arm
- the proportion of children with reduced parasite load (infection intensity) rates in the intervention and control arms

The secondary outcomes will be:

- the proportion of children with reduced prevalence of anemia in the intervention and control arms

Intervention Measures

Background: An unfortunate aspect of a nearly unique focus on large-scale anthelminthic drug administration was, and continues to be, the lack of concern within the global health community for prevention strategies, such as emphases on access to clean water, improved sanitation and hygiene activities that could serve as a backbone of multi-component integrated and sustainable disease control programs. Aiming at an integration and sustainability of parasite control program requires a package of intervention activities that are readily tailored to a given endemic setting and are fine-tuned over time. In this study, different intervention packages will be implemented considering the environment and the interrelated demographic, health and social systems contexts in the study area.

Hand Washing With Soap

High prevalence rates of intestinal parasites transmitted through faecal-oral route demonstrates the role played by unhygienic hands in transferring fecal particles from one host to another. Based on our preliminary survey, more than 89% of the children does not use soaps during hand washing and about 41% never wash their hands after defecation. These inadequate hand hygiene practices may transfer pathogens through direct interpersonal contact, contact with inanimate objects and surfaces, and during food preparations. Furthermore, in such setting, where the households store water they use for cooking and drinking in the house, dipping contaminated hands into storage containers can also transfer pathogens to other family members.

Hand-based transmission of pathogens is so ubiquitous that hand washing with soap has been argued to be the best intervention to prevent diarrhoea, and the most cost-effective option for prevention of morbidity and mortality in children.

Most people, especially children, do not wash their hands with soap at important times, such as after using toilet and before eating. The quantity and proximity of water available to households have been demonstrated to correlate with frequency of hand washing. Unfortunately, large proportion of people in developing countries do not have household-level access to piped water, which presents a formidable challenge to increase rates of hand washing with soap and water in such communities.

Methodology: Small bars of plain soap will be provided to each household with child in the hand washing with soap intervention and will be replenished as necessary. Children and family members in the respective group will be encouraged to wash their hands with water and soap before meals, after defecation, after playing on the ground and touching animals, before and after preparing food (mothers) and when ever their hands get unclean. Brief clarifications will be given on the clinical importance of intestinal parasitic infections and methods of prevention; proper hand washing procedures will be demonstrated during the commencement of the study and at every visit.

Training: Field workers will be trained for two days on the proper hand-washing with soap and nail clipping techniques, the purpose and procedures of the visits, supervisory activities, fellow-up, data collection, and reporting.

Study participants will be visited every week by data collectors and the investigator to determine compliance with hand hygiene, to answer any question, re-educate on the importance of hand hygiene and go over specific hand hygiene procedures.

Wash Your Hands: The Right Way

How to Wash

1. Wet hands and wrists

2. Apply soap

3. Lather for 45 seconds. Make sure you scrub in between fingers, the back of your hands, finger tips and wrists **(This is a VERY important step.)**

- A song related to hand-washing that the children sing in their school ‘let’s wash our hands with water and soap’ will be adjusted to fit the 45 seconds and children will be made to sing the song to keep the time needed to rub their hands.

4. Rinse

5. Dry your hands

- Wet your hands with clean, running water and apply soap
- Rub your hands together to make lather and scrub them well; be sure to scrub the backs of your hands, between your fingers, and under your nails.
- Continue rubbing your hands for at least 45 seconds. Need a timer? "Let's wash our hands with water and soap" song – the first beginning to end once.
- Rinse your hands well under running water.
- Dry your hands using a clean towel or air dry them.

When to Wash

- After using the toilet
- Before eating and feeding children
- Before, during, and after preparing food
- Before and after caring for someone who is sick
- After cleaning up a child who has used the toilet
- After touching an animal or animal waste
- After touching garbage
- After touching soil
- After blowing your nose, coughing, or sneezing

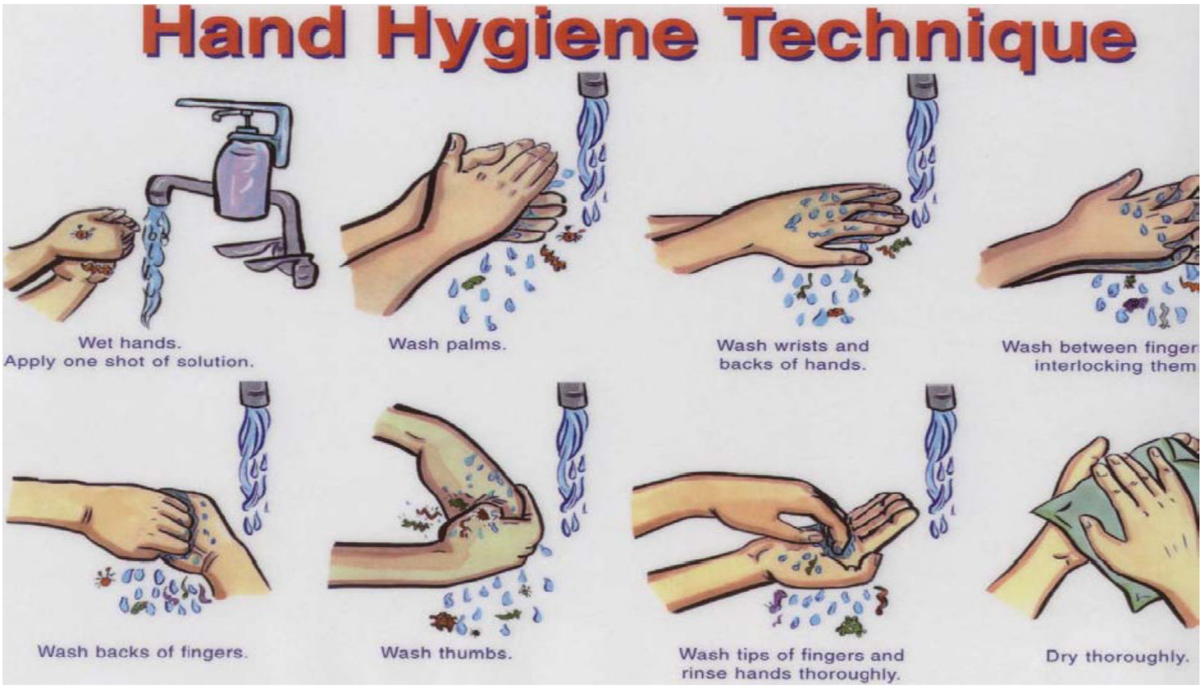

Fig. 1. **Steps to be followed during washing hands with soap**.

Hand Finger Nail Clipping

Studies have been able to isolate disease causing microorganisms from swabs of nails and nail clippings. Keeping nails clean and dry avoids collection of bacteria and other infectious organisms under the nails. Cutting nails short and washing hands by scrubbing nails after using the toilet and before meals has been shown to protect children and family from recurrence of parasitic infections. Preliminary data from our preliminary survey shows nail hygiene is of concern in school-aged children where 58.9 % of the children had unclipped finger nails and 49.1% of these nails were unhygienic.

Methodology: Children in the finger clipping intervention group will be provided with new nail clippers. Nail clippers will be kept by the field workers that will frequently visit the households to determine compliance with the intervention protocol, re-educate on the importance of nail trimming and go over specific nail clipping procedures. Each clipper will be kept tagged with codes for each child. Nail clipping will be performed by the health workers at weekly bases.

2.1. Procedure of nail clipping

- Cutting fingernails is easy. Just make sure you have a nice, sharp (**and above all safe**) nail cutter and a wastebasket to collect the nails
- Cut the nails straight across until they no longer extend over the finger, so that they don't show any white
- Follow the shape of the fingertip when trimming finger nails
- Do not cut the nails uneven, this may cause nails to split and crack and hence accumulate dirt and pathogens
- Look the nails over to ensure that they are all of the same length and shape, and also that they're smooth and not uneven or pointy. Keep clipping and filing until they all look the same and are smooth. Once they are, you're done!

Control condition: Control households will be provided with a regular monthly supply of sugar in an effort to preserve willingness to participate, but they will be given no products that would be expected to affect their hand-washing and nail clipping behaviours. They will be neither encouraged nor discouraged for hand-washing and nail clipping. Control households will be visited at equal frequency with the intervention households. Control

households will also frequently checked for the presence of soap for hand-washing and nail clippers during the weekly visits to check for contamination.

Sample collection and Analysis following 6 months intervention

Parasitological examination

- Following 6 months intervention children will be made to come to the schools were the laboratory will be established.
- A thumb sized fresh stool specimens will be collected on the spot from all the study subjects.
- Children will be provided with:
    - clean labelled plastic screw-top containers (for sample collection)
    - plastic sheet (to catch the stool in the toilet)
    - applicator stick (to transfer the sample).

Stool analysis will be done using:

- Direct saline wet mount
- Formalin ethyl-acetate concentration technique
- Kato-Katz technique (thick smear 41.7mg)

- Duplicate slides will be prepared for each stool specimen and for each of the techniques used.
- A child will be classified as re-infected if an infection was detected by any methods used.
- 10% of randomly selected stool smears will be re-examined for each sample collected for quality control purposes.

Haemoglobin survey

- At the end of the six months follow-up, haemoglobin concentration will be determined in finger prick blood using a HemoCue analyzer at the spot (HemoCue Hb 201z, Sweden).
– One day training of technicians collecting and analyzing blood samples
- Daily check of the HemoCue using reference microcuvettes based on the manufacturer instructions.
- Haemoglobin readings will be adjusted for altitude, and anemia will be defined for respective age and gender groups based on the World Health Organization cut-off values.

Data Processing and Analysis

Data will be entered and processed on an appropriate software and will be analysed using appropriate statistical analysis methods. Effectiveness of interventions will be assessed using regression models by odds ratio with help of SPSS version 16 software. Effectiveness of an intervention will be estimated based on a comparison of all individuals allocated to receive a particular intervention package versus those allocated not to receive the intervention. Infection intensity differences between intervention and control arms will be analysed by arithmetic mean differences for the egg counts using the independent-samples t-test. Participants will be analysed according to the group to which they were randomized. Base-line demographic and hygiene information will be obtained using structured questionnaire. Questionnaires will be coded by investigator and statistician, and data entry will be done using an appropriate statistical soft ware. Statistical significance will be tested with x^2 and shall be set at $p<0.05$.

Problems and pitfalls: The study is expected to have limitations arising from the study design to be followed, the process of data collection and nature of the study respondents. As behavioural trials are difficult to be blinded, systematic error is expected to occur due to the lack of blinding. An attempt to reduce this effect will be made by masking field workers to the identity of the assigned interventions and the outcome of interest. Field workers will check the presence of soap for hand-washing in the households other than those assigned to hand-washing intervention, and nail clippers in households other than assigned to the intervention during their weekly visits in an attempt to check for contamination.

Loss-to-follow-up will be reduced, and compliance during follow-up will be enhanced by recruiting children and households who are interested and reliable in to the selection process, and by arranging frequent contacts with individual members of the families.

9. Inclusion and exclusion criteria

Inclusion

- children who are able to bring signed written informed consent
- children / households who agree to participate throughout the study period
- children who permanently live in the area and who are going to stay in the house-hold throughout the study period
- children aged 6 to 15

Exclusion

- children who are not able to bring signed written informed consent
- children with known mental problems and sever physical handicap
- age less than 6 and above 15 years

Potential Risks

No major risks are expected to be associated with this study. Minor side effects like abdominal pain, nausea, vomiting and diarrhoea might be seen in heavily infested children following provision of treatment.

Ethical Consideration

Ethical clearance will be obtained from responsible bodies of Mekelle University and Tigray Regional Health Bureau. Linked confidential testing with informed written consent will be employed. A written assent will be obtained from the children's parents or guardians. Study participants will be informed using appropriate language and wording about the purpose of the study, anticipated benefits, how they are chosen to participate, data collection procedures and their full right to refuse, withdraw or completely reject part or all of the study. All formats containing results and other personal information will be kept locked and strictly confidential. Analysis results will be communicated only with relevant people.
