# Supplementary material for: Efficacy of Handwashing with Soap and Nail Clipping on Intestinal Parasitic Infections in School-Aged Children: A Factorial Cluster Randomized Controlled Trial
Source: PLoS Med. 2015 Jun 9;12(6):e1001837. doi: 10.1371/journal.pmed.1001837 (PMC4461173; doi:10.1371/journal.pmed.1001837)
Supplement: S1 Table — (DOCX) [file pmed.1001837.s004.docx]

**S1Table. Pre and post intervention prevalence of *E. histolytica* among school-aged children.**

|  | | **Post-intervention  *E. histolytica* prevalence** | | | **P-value^*^** |
| --- | --- | --- | --- | --- | --- |
|  |  | **No** | **Yes** | **Total** |  |
| **Pre-intervention  *E. histolytica* prevalence** | **No** | 230 | 27 | 257 | <0.0001 |
|  | **Yes** | 95 | 13 | 108 |  |
|  | **Total** | 325 | 40 | 365 |  |

^*^A McNemar test showed that the pre and post intervention *E. histolytica* prevalence change significantly.

*E. histolytica* = *Entamoeba histolytica*
