# Supplementary material for: Efficacy of Handwashing with Soap and Nail Clipping on Intestinal Parasitic Infections in School-Aged Children: A Factorial Cluster Randomized Controlled Trial
Source: PLoS Med. 2015 Jun 9;12(6):e1001837. doi: 10.1371/journal.pmed.1001837 (PMC4461173; doi:10.1371/journal.pmed.1001837)
Supplement: S3 Table — (DOCX) [file pmed.1001837.s006.docx]

**S3 Table. Pre and post intervention prevalence of Hookworm among school-aged children.**

|  | | **Post-intervention Hookworm prevalence** | | | **P-value^*^** |
| --- | --- | --- | --- | --- | --- |
|  |  | **No** | **Yes** | **Total** |  |
| **Pre-intervention Hookworm prevalence** | **No** | 335 | 4 | 339 | 0.001 |
|  | **Yes** | 21 | 5 | 26 |  |
|  | **Total** | 356 | 9 | 365 |  |

^*^A McNemar test showed that the pre and post intervention hookworm prevalence change significantly.
