# Supplementary material for: Efficacy of Handwashing with Soap and Nail Clipping on Intestinal Parasitic Infections in School-Aged Children: A Factorial Cluster Randomized Controlled Trial
Source: PLoS Med. 2015 Jun 9;12(6):e1001837. doi: 10.1371/journal.pmed.1001837 (PMC4461173; doi:10.1371/journal.pmed.1001837)
Supplement: S4 Table — (DOCX) [file pmed.1001837.s007.docx]

**S4 Table. Pre and post intervention prevalence of *E*. *vermicularis* among school-aged children.**

|  | | **Post-intervention  *E. vermicularis* prevalence** | | | **P-value^*^** |
| --- | --- | --- | --- | --- | --- |
|  |  | **No** | **Yes** | **Total** |  |
| **Pre-intervention  *E*. *vermicularis* prevalence** | **No** | 316 | 13 | 329 | 0.001 |
|  | **Yes** | 36 | 0 | 36 |  |
|  | **Total** | 352 | 13 | 365 |  |

^*^A McNemar test showed that the pre and post intervention *E. vermicularis* prevalence change significantly.

*E. vermicularis*=*Enterobius vermicularis*
